# Liver damage profile in COVID-19 pregnant patients

**DOI:** 10.1186/s12964-023-01285-z

**Published:** 2024-01-02

**Authors:** Marcin Januszewski, Laura Ziuzia-Januszewska, Michal Kudan, Kamil Pluta, Jakub Klapaczyński, Waldemar Wierzba, Tomasz Maciejewski, Alicja A. Jakimiuk, Artur J. Jakimiuk

**Affiliations:** 1 Department of Obstetrics and Gynecology, National Medical Institute of the Ministry of Interior and Administration, Wołoska 137, 02-507 Warsaw, Poland; 2 Department of Otolaryngology, National Medical Institute of the Ministry of Interior and Administration, Wołoska 137, 02-507 Warsaw, Poland; 3 Department of Hepatology, National Medical Institute of the Ministry of Interior and Administration, Wołoska 137, 02-507 Warsaw, Poland; 4 Department of Obstetrics and Gynecology, Institute of Mother and Child, Kasprzaka 17a, 01-211 Warsaw, Poland; 5 Department of Plastic Surgery, National Medical Institute of the Ministry of Interior and Administration, Wołoska 137, 02-507 Warsaw, Poland; 6 Center for Reproductive Health, Institute of Mother and Child, Kasprzaka 17a, 01-211 Warsaw, Poland

**Keywords:** COVID-19, Liver damage, Pregnancy, Bile acids, SARS-CoV-2

## Abstract

**Introduction:**

SARS-CoV-2 unsparingly impacts all areas of medicine. Pregnant women are particularly affected by the pandemic and COVID-19 related liver damage seems to be another threat to maternal and fetal health. The aim of this study is to define liver damage profile including bile acids serum levels in COVID-19 pregnant patients and to determine predictors of disease aggravation and poor obstetrics outcomes.

**Methods:**

This study has been carried out in the Obstetrics and Gynecology Department, at the National Medical Institute in Warsaw, Poland between 01.02.2021 and 01.11.2022 The study cohort comprises 148 pregnant patients with COVID-19 and 102 pregnant controls who has been tested negative for SARS-CoV-2.

**Results:**

COVID-19 pregnant patients presented liver involvement at admission in 41,9%. Hepatotoxic damage accounted for 27 (19.85%), cholestatic type was diagnosed in 11 (8.09%) and mixed type of liver injury was presented in 19 (13.97%) of patients. Higher serum levels of AST, ALT, GGT, total bilirubin and bile acids as well as mixed type of liver injury at admission were correlated with severe form of an illness. AST and ALT above upper reference limit as well as hepatotoxic type of liver damage predisposed pregnant patients with COVID-19 to poor obstetrics outcomes.

**Conclusion:**

Hepatic damage in pregnant women with COVID-19 is a common, mild, transaminase-dominant, or mixed type of injury, and often correlates with elevated inflammatory markers. SARS-CoV-2 test should be performed as a part of differential diagnosis in elevated liver function tests. Although bile acids serum levels were commonly elevated they seems to be clinically irrelevant in terms of pregnancy outcomes.

Video Abstract

**Supplementary Information:**

The online version contains supplementary material available at 10.1186/s12964-023-01285-z.

## Introduction

SARS-CoV-2 unsparingly impacts all areas of medicine. Pregnant women are particularly affected by the COVID-19 pandemic, requiring efficient preventative efforts. SARS-CoV-2 affects nearly every system, it`s extrapulmonary manifestations and long-term effects reflect magnitude of virus true extent. Novel coronavirus liver injury may base on several pathophysiological mechanisms [1]. Firstly, it may rely on direct cytopathic effect which is associated with SARS-CoV-2 potential to spread to different tissues due to its tropism to various determinants [2]. Angiotensin-2 Converting Enzyme (ACE2) receptor is present on both hepatocytes (in 2,6% of cells) and cholangiocytes (in 59,7% of cells) [3]. Moreover, essential for attacking host system co-receptors: transmembrane serine protease 2 (TMPRSS2) and furin-like proteases are highly expressed in hepatic cells [4]. Secondly, the dysregulation of immune system reflected in increased levels of inflammatory markers can cause liver injury [5]. Activation of immune cells and cytokine storm including Tumor Necrosis Factor-alpha (TNF‐alpha), Interleukin-1 (IL‐1) and Interleukin-6 (IL‐6) coordinates the innate immune response to tissue damage and liver cell death in the sterile inflammation process [6].

Additionally, there have been reports of a two-phase pattern showing initial transaminase elevations followed by cholestatic liver enzymes [7, 8]. This process can be manifested in increased bile acids levels in serum. Another potential mechanism includes hypoxic alterations in liver cells resulting from circulatory and respiratory failure [9]. Finally, drug-induced liver injury caused by novel treatment with hepatotoxic substances plays an important role in worsening liver damage during hospitalization.

Liver adaptation to pregnancy comprises its enlargement, changes in metabolic function and hepato-biliary system [10]. In pregnancy high serum level of estrogen and progesterone especially in third trimester induces in liver pro-cholestatic state [10, 11]. Furthermore, during pregnancy modulation in the immune defense system occurs from anti-inflammatory state in second trimester to pro-inflammatory condition in third trimester. This physiological adaptation in combination with the hypercoagulability creates predisposition of SARS-CoV-2 infected pregnant patients to more severe course of an illness [12, 13]. Additionally, COVID-19 in pregnancy is characterized by higher levels of inflammatory markers [14].

### Aim of the study

The objective of our study was to define a liver damage profile in COVID-19 positive pregnant patients. The secondary objective of our study was to indicate which abnormal laboratory liver function test is correlated with the inflammation markers and disease severity. For patients who delivered within 2 weeks of hospital admission we attempted to determine which liver function test correlated with poor obstetric outcome measured as incidence of preterm birth, birth weight < 2500 g, percentile of birth weight < 10, APGAR scores at 0 and 5 min < 8 and the occurrence of green amniotic fluid.

## Materials and methods

### Study population

This prospective single-centered case–control study was undertaken in the Department of Obstetrics and Gynecology, at the Central Clinical Hospital of the Ministry of the Interior and Administration in Warsaw, Poland between 01.02.2021 and 01.11.2022. The study group consists of 148 COVID-19 pregnant patients admitted to hospital for treatment, labor, or indication for induction of labor. The control group was 102 SARS-CoV-2 negative pregnant patients admitted to hospital for obstetrics indications. The study cohort and control groups were recruited continuously due to the seasonality of ICP. The research received approval from the Bioethics Committee at the Central Clinical Hospital of the Ministry of the Interior and Administration in Warsaw (decision number 17/2020) and conforms to the provisions of the Declaration of Helsinki. All the patients signed informed written consent prior to the participation.

### COVID-19 confirmation and laboratory assays

To validate SARS-CoV-2 status nasopharyngeal swab for PCR or antigen test were taken on admission. The N, E, and RdPd genes were subjected to gene amplification through reverse transcriptase real-time polymerase chain reaction (rRT-PCR). The detection kit used for the assay was the GeneProof SARS-CoV-2 PCR Kit from GeneProof a.s., Brno, Czech Republic, following the protocols provided by the kit manufacturer. We employed rapid antigen tests that fulfilled the diagnostic requirements with a sensitivity of ≥ 80% and a specificity of ≥ 97%. Those tests included the Panbio™ COVID-19 AG Rapid Test Device from Abbott, Abbot Rapid Diagnostics Jena GmbH, Jena, Germany, and the Bioeasy 2019-nCoV Ag Fluorescence from Shenzhen Bioeasy Biotechnology Co. Ltd., Shenzhen, China.

Laboratory biochemistry assays were performed at Cobas 8000 System (Roche Diagnostics, Basel, Switzerland). Urine analyses were performed at URiSCAN PlusScope from (YD diagnostics, Yongin, South Korea), complete blood count at Sysmex XN analyser (Sysmex, Kobe, Japan) and coagulation profile at ACL TOP 500 analyser (Werfen UK, Warrington, UK).

### Progression of the disease and therapeutic management

Patients were divided into 4 stages of clinical aggravation, following the Polish Association of Epidemiologists and Infectiologists Guidelines, including mild, moderate, severe and critical courses of the illness [15]. The mild course was characterized by asymptomatic or presence of mild symptoms, moderate by radiological or clinical lung occupation, severe by respiratory failure and critical by acute respiratory distress syndrome (ARDS), multiorgan failure, hypotensive shock or the need for mechanical ventilation. Patients were treated according to national guidelines [15].

### Study procedures

On admission, all patients underwent complete blood tests, urine test, coagulation profile and biochemical test including liver function tests (LFTs) including Bile Acids serum concentration (BA). When clinical presentation or lab tests indicated moderate, severe, or critical form of illness, CT chest scan was performed. We excluded patients with history of known liver diseases, cholelithiasis (by ultrasound) and diagnosed with preeclampsia (by abnormal Soluble fms-like tyrosine kinase-1/ Placental Growth Factor ratio (sFLt-1/PLGF ratio), HELLP and ICP (by the presence of pruritis no other explained) during hospitalization. There were no patients with the diagnosis of AFLP. All the pregnant patients underwent screening tests first and third trimester for liver infectious diseases according to national criteria: Hepatitis B and C, HIV, Toxoplasma gondii and Rubella [16]. There were no patients with disordered thyroid disease, history of drug or alcohol abuse and liver transplantation. Treatment protocol for COVID-19 infection requires usage of drugs affecting liver function including antipyretic drugs (paracetamol, ibuprofen), antiviral drugs (Remdesivir), antibiotics (Ceftriaxon, Azitromycin) and Low Molecular Weight Heparin (LMWH). Therefore, we decided to analyze based on admission data, most precisely reflecting liver damage in COVID-19 pregnant patients.

After these procedures 237 patients for final analyses left divided into SARS-CoV-2 positive (136 patients) and 101 SARS-CoV-2 negative group.

### Liver test abnormalities

Liver biochemical test abnormalities can be grouped into 3 classic patterns: hepatocellular (when disproportionate elevation in the serum aminotransferases compared with the alkaline phosphatase occurs), cholestatic (disproportionate elevation in the alkaline phosphatase compared with the serum aminotransferase), isolated hyperbilirubinemia (elevated bilirubin level with normal serum aminotransferases and alkaline phosphatase) and mixed (the liver test abnormalities are characterized by the predominant abnormality). However, consensus on the liver damage classification due to SARS-CoV-2 infection is scarce. Regarding non-pregnant cohort hepatocellular, cholestatic and mixed patterns were proposed [17].

Liver damages were described as the elevation of following enzymes in serum:

Aspartate aminotransferase (AST) > 31 U/l, Alanine aminotransferase (ALT) > 31 U/l Gamma-glutamyltransferase (GGT) > 36 U/l total bilirubin (TBIL) > 1,2 mg/dl, Alkaline phosphatase (ALP) > 104 U/l Bile acids (BA) > 10 µmol/l. Due to placental production of ALP, and its elevated serum levels especially in 3rd trimester ALP levels are not considered to be appropriate indicator of cholestatic liver damage in pregnant cohort. Therefore, we decided to perform bile acids serum test as an indicator of cholestasis.

We categorized the COVID-19 abnormalities into hepatocellular, cholestatic, or mixed patterns. Because most patients presented mild and moderate magnitude of the elevation of liver test values, we defined the patterns of liver abnormalities according to following criteria:

Hepatocellular pattern was described when AST and/or ALT serum levels were above upper reference limit (URL). Additionally, 3xURL in that group were considered as severe hepatocellular damage.

Cholestatic pattern was recognized when BA and/or GGT serum levels were above URL. Additionally, 2 × URL in that group was classified as severe cholestatic damage. Mixed type occurred when AST and/or ALT serum levels were above URL and BA and/or GGT serum levels were above URL.

### Statistical analysis

Statistical analysis was performed using Statistica software (version 13.3; StatSoft, Poland). We considered a two-sided *p*-value of less than 0.05 to indicate statistical significance. As most of the continuous variables were non-normally distributed, they were reported as median and interquartile range (IQR) and compared with a Mann–Whitney U-test. Categorical variables were presented as the number of patients and percentages and compared with the chi-squared test with Yates correction or Fisher’s exact test as appropriate. Comparisons of the liver function biomarkers between the three disease COVID-19 severity groups were performed using the Kruskal–Wallis test followed by pair-wise comparison using Dunn’s post hoc test for continuous variables, and Pearson’s chi-square test for categorized variables. The association between the inflammatory biomarkers’ levels and the elevation of liver injury biomarkers was analyzed using a Mann–Whitney U-test. Univariate logistic regression analyses were performed to determine the association of the type of liver damage and the risk of progression to severe course of COVID-19, the association of the type of liver damage and poor obstetric outcomes, as well as the association of the elevation of liver injury biomarkers and poor obstetric outcomes. We used the Weight of Evidence tool to fill in the missing values. For the association of the elevation in liver injury biomarkers with poor obstetric outcomes, we incorporated variables that showed significance in the univariate analysis into a stepwise multivariate logistic regression.

## Results

In the study group the median (IQR) of maternal age was 32 (30;36), the median (IQR) body mass index (BMI) was 27,76 (24.54; 31.8) kg/m2. with median (IQR) gestational age at admission 35 (29.5; 39) weeks ranging from 18 to 41 weeks. 116 (85, %) of patients were in 3^rd^ trimester presenting gastroenterological symptoms—nausea or vomiting in 3 (2,21%) and diarrhea in 3 (2,21%) patients. There were no significant differences between the groups concerning maternal age, BMI, presence of diabetes, hypertension, and hypothyroidism. However, thrombophilia, pregnancies after IVF, and more advanced gestational weeks were more common in the control group. These confounding factors might have led to an underestimation of liver damage prevalence in SARS-CoV-2 positive patients, rather than the opposite. Ninety-six (70,59%) patients were classified as a mild course of illness. Moderate and severe COVID-19 accounted for 14 (10,29%) and 26 (19,12%) cases respectively. No critical course of an illness was diagnosed.

COVID-19 pregnant patients in comparison with SARS-CoV-2 negative counterparts presented at admission higher levels of liver injury markers with exception of ALP (Table 1). 26 patients (19.85%) had bile acid > 10 µmol/l and 3 above 40 µmol/l which was considered as highly fetotoxic [18]. SARS-CoV-2 infected patients presented at admission: AST > URL in 38 (28,79%) patients with 6 patients (4,55%) > 3 URL. ALT above upper limit of normal was present in 36 (27,07%) patients with 6 (4,51%) > 3 URL. 10 (4,22%) of patients had GGT > URL. ALP > URL was presented in 97 (74,5%) with 4 (3,05%) > 3 URL. Six patients (4,62%) and 2 (1,54%) had > URL and > 3 URL of TBIL serum levels. No patients presented > 10 URL of any liver damage marker level at initial presentation and therefore no cases of severe liver failure have been observed. Patients’ characteristics, clinical, obstetrics and laboratory results are presented in Table 1.

**Table 1:** Patients characteristics, clinical, obstetrics and laboratory results

Parameter		Total (*N* = 237)	COVID-19 patients (*N* = 136)	Controls (*N* = 101)	*p*-value*
Maternal age [years] (*N* = 237)		30 (30; 36)	32 (30; 36)	33 (30; 37)	0.148*
Maternal BMI [kg/m2] (*N* = 213)		27.64 (25.01; 31.55)	27.76 (24.54; 31.8)	27.64 (25.73; 31.25)	0.873*
Gestational age at admission [weeks] (*N* = 237)		38 (32; 39)	35 (29.5; 39)	39 (36; 39)	0.004*
Gestational age at admission; N (%)	18–22 [weeks]	6 (2,53%)	5 (3,7%)	1 (0.99%)	
	23–27 [weeks]	21 (8,86%)	15 (11%)	6 (5,9%)	
	28–33 [weeks]	46 (19,41)	35 (25,7%)	11 (10,9%)	
	34–36 [weeks]	28 (11,81%)	19 (13,9%)	9 (8,9%)	
	37–41 [weeks]	136 (57,38%)	62 (45,6%)	74 (73,3%)	
Length of hospitalization [days] (*N* = 237)		5 (4; 7)	5 (3; 7)	5 (5; 6)	0.004*
COVID-19 severity; N (%)	1		96 (70.59%)		
	2		14 (10.29%)		
	3		26 (19.12%)		
	4		0(0%)		
Percentage of lung involvement on CT; Median (IQR); (*N* = 45)			20 (10; 31)		
IVF (*N* = 237)		20 (8.44%)	1 (0.74%)	19 (18.81%)	< 0.001**
Any comorbidities		119 (50.21%)	59 (43.38%)	60 (59.41%)	0.021***
Thrombophilia		10 (4.22%)	2 (1.47%)	8 (7.92%)	0.020**
Diabetes		44 (18.57%)	24 (17.65%)	20 (19.8%)	0.800***
Hypertension		12 (5.06%)	4 (2.94%)	8 (7.92%)	0.132**
Hypothyroidism		67 (28.27%)	32 (23.53%)	35 (34.65%)	0.083***
Bile acids at admission (*N* = 231)		4 (2; 7)	5 (3; 9)	3 (2; 5)	< 0.001*
Bile acids – maximum level (*N* = 232)		4 (2; 7)	5 (3; 9)	3 (2; 5)	< 0.001*
Bile acids at admission > 10 (*N* = 231)		31 (13.42%)	26 (19.85%)	5 (5%)	< 0.001**
Bile acids at admission > 40 (*N* = 231)		4 (1.73%)	3 (2.29%)	1 (1%)	0.635**
Bile acids max > 10 µmol/l (*N* = 232)		32 (13.79%)	27 (20.45%)	5 (5%)	< 0.001**
Bile acids max > 40 µmol/l (*N* = 232)		4 (1.72%)	3 (2.27%)	1 (0.43%)	0.636**
ALP at admission (*N* = 225)		142 (107; 187)	144 (104; 194)	140 (108; 164)	0.389*
ALP at admission > URL (*N* = 225)		172 (76.44%)	97 (74,.05%)	75 (79.79%)	0.400***
ALP at admission > 3 × URL (*N* = 225)		5 (2.22%)	4 (3.05%)	1 (1.06%)	0.403***
ALT at admission (*N* = 231)		15 (11; 25)	17 (11; 34)	13 (10; 19)	0.001*
ALT at admission > URL (*N* = 231)		44 (19.05%)	36 (27.07%)	8 (8.16%)	< 0.001***
ALT at admission > 3 × URL (*N* = 231)		7 (3.03%)	6 (4.51%)	1 (1.02%)	0.243**
APTT at admission (*N* = 231)		29.4 (26.9; 33.1)	32.1 (28.55; 35.6)	27.4 (25.4; 29.7)	< 0.001*
APTT at admission > URL (*N* = 231)		24 (10.39%)	24 (18.18%)	0 (0%)	< 0.001**
PT at admission (*N* = 230)		10.65 (10.2; 11)	10.5 (10.1; 10.9)	10.8 (10.3; 11.1)	0.024*
PT at admission > URL (*N* = 230)		4 (1.74%)	4 (3.05%)	0 (0%)	0.136**
INR at admission > URL (*N* = 231)		3 (1.3%)	3 (2.26%)	0 (0%)	0.264**
AST at admission (*N* = 230)		20 (16; 28)	23 (18; 34.5)	18 (14; 20)	< 0.001*
AST at admission > URL (*N* = 230)		45 (19.57%)	38 (28.79%)	7 (7.14%)	< 0.001***
AST at admission > 3 × URL (*N* = 230)		6 ((2.61%)	6 (4.55%)	0 (0%)	0.039**
Total bilirubin at admission (*N* = 222)		0.3 (0.21; 0.45)	0.35 (0.25; 0.5)	0.26 (0.19; 0.35)	< 0.001*
Total bilirubin at admission > URL (*N* = 222)		7 (3.15%)	6 (4.62%)	1 (1.09%)	0.244**
Total bilirubin at admission > 3 × URL (*N* = 222)		2 (0.9%)	2 (1.54%)	0 (0%)	0.512**
Bilirubin in urinalysis at admission (*N* = 141)		1 (0.71%)	1 (1.04%)	0 (0%)	1**
Urobilinogen in urinalysis at admission (*N* = 141)		4 (2.84%)	3 (3.16%)	1 (2.17%)	1**
GGT at admission (*N* = 231)		10 (7; 17)	11 (8; 22)	8 (6; 12)	< 0.001*
GGT at admission > URL (*N* = 231)		11 (54.76%)	10 (4.22%)	1 (0.43%)	0.027**
GGT at admission > 3 × URL (*N* = 231)		0 (0%)	0 (0%)	0 (0%)	1**
Total amylase at admission (7)		72 (63; 87)	94 (72; 116)	64 (63; 77)	0.333*
CRP at admission (*N* = 229)		6.1 (3; 22.5)	12.6 (4.8; 38.8)	3.6 (2; 6.5)	< 0.001*
LDH at admission (*N* = 142)		199.5 (170; 243)	204 (171; 249)	175 (164; 198.5)	0.061*
WBC at admission (*N* = 235)		9.46 (7.68; 11.49)	8.57 (6.85; 10.43)	10.55 (9.12; 12.43)	< 0.001*
PLT at admission (*N* = 234)		200 (170; 240)	191 (161; 221)	209 (187; 254)	< 0.001*
NEU at admission (*N* = 235)		7.16 (5.62; 8.86)	6.43 (5.1; 8.28)	7.8 (6.6; 9.5)	< 0.001*
LYMPH at admission (*N* = 235)		1.5 (1.08; 1.98)	1.24 0.92; 1.7)	1.81 (1.4; 2.24)	< 0.001*
PCT at admission (*N* = 136)		0.08 (0.05; 0.15)	0.08 (0.05; 0.14)	12.19 (10.01; 13.87)	0.916*
INR at admission (*N* = 231)		0.97 (0.93; 1)	0.96 (0.92; 0.99)	0.98 (0.94; 1)	0.067*
IL-6 at admission (*N* = 130)		14 (5.39; 28)	14.3 (5.39; 28)	13 (13; 13)	1*

^*^Mann–Whitney test. Data are presented as median (IQR)

^**^Fisher exact test. Data are presented as N (%)

^***^Chi-square test with Yates correction. Data are presented as N (%)

In the study group fifty-four pregnant patients presented liver damage at admission (41,9%). Hepatotoxic damage accounted for 27 (19.85%) patients with 23(16,91%)of them having mild (> URL) and 4 (2.94%) severe type (> 3 URL). Cholestatic type was diagnosed in 11 (8.09%) including severe form in 3 (2.21%) patients. Mixed type of liver injury presented 19 (13.97%) patients. TBIL in 6 cases was not separately exceeding URL and thus no isolated hyperbilirubinemia was diagnosed. Classification patterns of liver abnormalities are presented in Table 2.

**Table 2:** Types of liver damage

**Type of liver damage**	**Overall N (%)**	Non-severe **N (%)**	Severe **N (%)**	Progression to Severe Course of illness^*^		
				OR	95%CI	*p*-value*
Cytotoxic	27 (19.85%)	23(16,91%)	4 (2.94%)	2.18	0.82;5.78	0.117
Cholestatic	11 (8.09%)	8(5,88%)	3 (2.21%)	0.40	0.05;3.31	0.399
Mixed overall	19 (13.97%)			5.46	1.92;15.5	0.001

^*^Univariate logistic regression

Among COVID-19 pregnant patients AST, ALT and GGT > URL were positively correlated with inflammatory parameters (PCT, CRP and IL-6) and negatively with white blood count. AST > URL at admission correlated positively with lung involvement on CT chest scan. Liver function tests association with inflammatory markers are presented in Table 3.

**Table 3:** Liver function tests association with inflammatory markers

**Parameter**	**bile acids at admission > 10**		*p*-value*
	**Yes (*N* = 26)**	**No (*N* = 105)**	
Percentage of lung involvement on CT (*N* = 45)	21 (16; 30.5)	18.5 (5; 30)	0.452
CRP at admission (*N* = 133)	15.7 (4.6; 68.1)	12.4 (4.8; 32.3)	0.334
WBC at admission (*N* = 146)	7.35 (5.62; 9.48)	8.67 (7.25; 10.69)	0.015
PCT at admission (*N* = 130)	0.11 (0.07; 0.26)	0.08 (0.05; 0.13)	0.057
IL-6 at admission (*N* = 129)	18.5 (8.14; 50.8)	13.5 (5.15; 26.8)	0.061
**Parameter**	**GGTP at admission > URL**		*p*-value*
	**Yes (*N* = 10)**	**No (*N* = 123)**	
Percentage of lung involvement on CT (*N* = 45)	30 (26; 35.5)	17 (6; 30)	0.080
CRP at admission (*N* = 133)	37.8 (25.3; 137.5)	12.1 (4.6; 36.5)	0.004
WBC at admission (*N* = 146)	5.98 (4.82; 7.85)	8.63 (7.1; 10.72)	0.009
PCT at admission (*N* = 130)	0.25 (0.13; 0.58)	0.08 (0.05; 0.13)	< 0.001
IL-6 at admission (*N* = 129)	36.3 (24.1; 54.3)	13.4 (5.2; 26.8)	0.005
**Parameter**	**ALP at admission > URL**		*p*-value*
	**Yes (*N* = 97)**	**No (*N* = 34)**	
Percentage of lung involvement on CT (*N* = 45)	22 (13; 37)	18.5 (1; 32.5)	0.336
CRP at admission (*N* = 133)	12.1 (4.6; 36.5)	18.9 (5.8; 44.7)	0.473
WBC at admission (*N* = 146)	8.61 (7.02; 10.4)	8.11 (5.9; 9.83)	0.303
PCT at admission (*N* = 130)	0.08 (0.05; 0.16)	0.08 (0.04; 0.13)	0.460
IL-6 at admission (*N* = 129)	12.95 (5.39; 27.1)	18.5 (4.62; 39.8)	0.629
**Parameter**	**ALT at admission > URL**		*p*-value*
	**Yes (*N* = 36)**	**No (*N* = 97)**	
Percentage of lung involvement on CT (*N* = 45)	30 (18.5; 40)	15 (6; 30)	0.085
CRP at admission (*N* = 133)	26.25 (9.4; 67.7)	10.65 (4.2; 31.15)	0.007
WBC at admission (*N* = 146)	7.39 (5.67; 10.14)	8.67 (7.32; 10.76)	0.023
PCT at admission (*N* = 130)	0.14 (0.08; 0.31)	0.07 (0.05; 0.12)	< 0.001
IL-6 at admission (*N* = 129)	16.5 (8.07; 45)	13.3 (5.15; 24.7)	0.035
**Parameter**	**AST at admission > URL**		*p*-value*
	**Yes (*N* = 38)**	**No (*N* = 94)**	
Percentage of lung involvement on CT (*N* = 45)	30 (17; 40)	15 (5; 25)	0.004
CRP at admission (*N* = 133)	36.7 (19.5; 95)	8.2 (3.8; 25.3)	< 0.001
WBC at admission (*N* = 146)	7.87 (5.78; 9.97)	8.65 (7.25; 10.85)	0.049
PCT at admission (*N* = 130)	0.19 (0.12; 0.31)	0.06 (0.04; 0.09)	< 0.001
IL-6 at admission (*N* = 129)	30.9 (13.6; 54.6)	0.51 (4.48; 19.4)	< 0.001
**Parameter**	**bilirubin at admission > URL**		*p*-value*
	**Yes (*N* = 6)**	**No (*N* = 124)**	
Percentage of lung involvement on CT (*N* = 45)	20 (20; 30)	20 (7; 31)	0.605
CRP at admission (*N* = 133)	35.55 (25.3; 108)	12.5 (4.75; 38.95)	0.062
WBC at admission (*N* = 146)	8.71 (7.97; 10.49)	8.52 (6.77; 10.39)	0.727
PCT at admission (*N* = 130)	0.19 (0.13; 0.31)	0.08 (0.05; 0.14)	0.076
IL-6 at admission (*N* = 129)	37.5 (24.1; 61.8)	13.6 (5.31; 27.1)	0.018

^*^Mann–Whitney test. Data are presented as median (IQR)

Higher serum levels of AST, ALT, GGT, TBIL, APTT, INR, and maximum serum levels of BA predisposed COVID-19 pregnant women to severe course of an illness. Moreover, initial serum level > URL of BA, AST, GGT, total bilirubin as well as mixed type of liver injury at admission was correlated with disease aggravation (Tables 2 and 4).

**Table 4:** Liver function tests in correlation with course of COVID-19 severity

Parameter	Course severity of COVID 19				*p*-value*
	**1 (*N* = 96)**	**2 (*N* = 14)**	**3 (*N* = 26)**	**H**	
Bile acids at admission (*N* = 131)	5 (3; 9)	5.5 (3; 10)	6.5 (4; 19.5)	4.4	0.111
Bile acids – maximum level (*N* = 132)	5 (3; 9)	5.5 (3; 10)	7 (5; 20)	6.87	0.032 (p^1/3^ = 0.027)*
Bile acids at admission > 10 (*N* = 131)	14 (15%)	3 (21%)	9 (38%)		0.048**
ALT at admission (*N* = 133)	15 (11; 30)	13 (12; 15)	26 (17; 59)	9.92	0.007 (p^1/3^ = 0.0154; p^2/3^ = 0.023)*
ALT at admission > URL	23 (24%)	2 (14%)	11 (44%)		0.078**
AST at admission (*N* = 132)	22 (18; 29)	20.5 (17; 30)	38 (30; 61)	17.01	< 0.001 (p^1/3^ < 0.001; p^2/3^ = 0.016)*
AST at admission > URL	18 (19%)	3 (21%)	17 (68%)		< 0.001**
GGTP at admission (*N* = 133)	10 (7; 19)	13 (9; 30)	23 (12; 35)	16.87	< 0.001 (p^1/3^ < 0.001)*
GGTP at admission > URL	2 (2%)	2 (14%)	6 (24%)		< 0.001**
ALP at admission (*N* = 131)	161.5 (118; 208.5)	124 (99; 169)	112 (92; 136)	13.36	0.001 (p^1/3^ = 0.002)*
ALP at admission > URL	75 (82%)	8 (62%)	14 (54%)		0.010**
Total bilirubin at admission (*N* = 130)	0.32 (0.23; 0.46)	0.33 (0.24; 0.48)	0.48 (0.34; 0.71)	9.82	0.007 (p^1/3^ = 0.005)*
Total bilirubin at admission > URL	1 (1%)	2 (14%)	3 (12%)		0.013**
APTT at admission (*N* = 132)	30 (27.5; 34.4)	34 (31.7; 36.8)	35.8 (32.8; 38.5)	18.67	< 0.001 (p^1/2^ = 0.025; p^1/3^ < 0.001)*
APTT at admission > URL (*N* = 132)	11 (12%)	3 (21%)	10 (40%)		0.005**
INR at admission (*N* = 133)	0.96 (0.91; 0.98)	0.98 (0.9; 1.01)	0.98 (0.96; 1.03)	7.8	0.020 (p^1/3^ = 0.016)*
INR at admission > URL (*N* = 133)	2 (2%)	0 (0%)	1 (4%)		0.714**
PT at admission (*N* = 131)	10.5 (10; 10.8)	10.75 (9.9; 11.1)	10.8 (10.4; 11.2)	1.04.1979	0.091*
PT at admission > URL (*N* = 131)	4 (4%)	0 (0%)	0 (0%)		0.417**

Data are presented as median (interquartile ranges). *P* values were calculated using the Kruskal–Wallis test (df = 2 for all variables) followed by Dunn’s post hoc test. p1/2 values indicate the comparison between course severity 1 (mild disease) and 2 (moderate disease); p1/3 values indicate the comparison between course severity 1 (mild disease) and 3 (severe disease); p2/3 values indicate the comparison between course severity 2 (moderate disease) and 3 (severe disease)

^*^Kruskall-Wallis test + post-hoc Dunn test

^**^chi-square test

Univariate logistic regression revealed that AST > URL, ALT > URL and hepatotoxic type of liver damage predisposed COVID-19 pregnant patients to poor obstetrics outcomes measured in incidence of preterm birth, and birth weight < 2500 g. Additionally AST > URL and hepatotoxic type of liver injury increased the probability of low APGAR score at 1 min and green amniotic fluid incidence (Tables 5 and 6).

**Table 5:** Obstetric outcomes

Variable	Preterm birth			Neonatal birth weight < 2500			Percentile of birth weight < 10		
	OR	95%CI	*p*-value	OR	95%CI	*p*-value	OR	95%CI	*p*-value
Bile acids > 10	1.97	0.65; 5.97	0.230	1.64	0.46; 5.9	0.445	0.86	0.09; 8.07	0.894
ALT > URL	3.05	1.07; 8.63	0.036	3.4	1.04; 11.13	0.043	0.55	0.06; 5.09	0.596
AST > URL	7.89	2.61; 23.89	< 0.001	7.82	2.19; 27.96	0.002	4.17	0.66; 26.3	0.129
GGT > URL	2.77	0.62; 12.29	0.181	2.26	0.416; 12.27	0.345	2.94	0.29; 29.51	0.360
Cytotoxic	4,29	1.44;12.758	0.009	6,14	1.81;20.86	0.004	2,73	0.43;17.47	0.288
Cholestatic	-	-	0.99	-	-	0.997	-	-	0.997
Mixed type	2,47	0.75;8.17	0.14	1,71	0.42;7.02	0.4536	1,34	0,14;12,81	0.797

Univariate logistic regression

**Table 6:** Obstetrics outcomes

Variable	Apgar on 1 min < 8			Apgar on 5 min < 8			Green amniotic fluid		
	OR	95%CI	*p*-value	OR	95%CI	*p*-value	OR	95%CI	*p*-value
Bile acids > 10	2.62	0.69; 9.97	0.157	0.67	0.07; 6.03	0.721	0.22	0.03; 1.86	0.166
ALT > URL	3.36	0.94; 11.98	0.062	5.41	0.94; 31.24	0.059	0.69	0.2; 2.42	0.560
AST > URL	8.85	2.16; 36.24	0.002	-	-	-	2.05	0.66; 6.41	0.217
GGT > URL	2.79	0.5; 15.52	0.240	2.3	0.24; 22.16	0.471	-	-	-
Cytotoxic	5,93	1.6;21.8	0.0074	-	-	-	3,58	1.08;11.89	0.038
Cholestatic	1,26	0.14;11.29	0.838	-	-	0.997	0,51	0.057;4.57	0.547
Mixed	2,17	0.51;9.19	0.293	-	-	0.965	-	-	0.996

Univariate logistic regression

In multivariate logistic regression patients with AST > URL were at a 7-fold greater risk of preterm birth: (OR 7.89, 95%CI 2.61; 23.89, *p* < 0.001), at a 8-fold greater risk of 1’APGAR score < 8 (OR 8.85, 95%CI 2.16; 36.24, *p* = 0.002) and at 7-fold greater risk of neonatal birth weight < 2500g (OR 7.82,95%CI 2.19; 27.96, *p* = 0.002).

At initial presentation SARS-CoV-2 infected pregnant women with mixed type of liver injury were more prone to disease progression toward greater clinical severity and higher levels of inflammation biomarkers. Cytotoxic type of hepatic disfunction however predisposed to poor obstetric outcome.

## Discussion

### Liver test abnormalities and types of damage

To our knowledge our study is the first describing liver function abnormalities with the use of bile acids serum levels and types of liver damage among pregnant patients infected with SARS-CoV-2.

Our study indicated that COVID-19 pregnant patients in comparison with healthy counterparts presented at admission higher serum levels of AST, ALT, GGT, bile acids and total bilirubin. Any abnormality in LFTs excluding ALP were present in 41,9%, rarely exceeding 3 × URL. In the study made by Cai Q et al., at initial presentation any abnormality was diagnosed in nearly 50% of non-pregnant patients with 5% exceeding 3 × URL for hepatic and 2 × cholestatic injury markers [17]. In our study elevation of AST, ALT and bile acids in 38 (28.79%), 36 (27.07%), 27 (20.45%) was the most frequently occurring abnormality. GGT levels exceeded URL only in 10 (4.22%) patients with no > 3 URL cases. In the general population, aminotransferases at admission were elevated at similar percentage of patients with the exception of GGT, which abnormal results at admission appeared to be more pronounced, with 58 (13.91%) having > URL and 10 (2.4%) having more than 3 × URL [17]. In our study, two groups presented similar ALP serum levels, which confirms expected poor diagnostic value of this parameter in pregnant patients.

In pregnant group of patients, LFTs measurements performed during hospitalization indicated higher prevalence of abnormal results and aggravation of liver damage than in non-pregnant COVID-19 positive control group were outlined in works of Li Q et al. and Chen H et al. [19, 20]. In those works COVID-19 probably exacerbated underlying obstetric conditions associated with liver injury, such as preeclampsia, HELLP or intrahepatic cholestasis of pregnancy.

In our study hepatotoxic damage accounted for 27 (19.85%) with 23(16,91%) had mild (> URL) and 4 (2.94%) severe type (> 3 URL). Cholestatic type was diagnosed in 11 (8.09%) including severe form in 3 (2.21%) patients and mixed type of liver injury presented 19 (13.97%) patients. In pregnant patients COVID-19-associated liver AST-dominant hepatic type injury largely mirrored non-pregnant cohort [20–23]. Those studies however were characterized by small sample or excluded cholestatic LFTs from analysis. It seems that bile acids levels may show cholestatic liver damage most accurately in pregnant group of COVID-19 patients.

High levels of bile acids > 40 µmol/l, potentially fetotoxic without the presence of pruritis occurred in 3 (2.29%) of patients. High levels of bile acids, associated with fetal mortality strongly influenced decision regarding delivery. Bile acids serum levels measurements may have serious clinical significance in this group of patients.

### LFTs in correlation with inflammatory markers

Among COVID-19 pregnant patients AST, ALT and GGT > URL were positively correlated with inflammatory parameters (PCT, CRP and IL-6) and negatively with white blood count. AST > URL at admission correlated positively with lung involvement on CT chest scan.

SARS-CoV-2 is associated with a spontaneous immune response with increased concentrations of several clinically evaluated inflammatory biomarkers including CRP, PCT and IL-6. Severe COVID-19 infection induces systemic inflammation called a “cytokine storm”, characterized by exaggerated production of soluble immune mediators and is responsible for tissue damage in course of COVID-19 [24, 25]. Moreover, COVID-19 in pregnancy, as a pro-inflammatory state is associated with higher levels of inflammatory biomarkers [14]. These results suggest that elevated inflammatory cytokines are the most significant feature and predictor of severe COVID-19, leading to liver damage in these patients. Therefore CRP, PCT and IL-6 are more pronounced in COVID-19 with liver injury [24, 26] described by characteristic pattern of subsequent relation in work of Diaz-Louzao et al. [27].

### LFTs and disease progression

Higher levels of AST, ALT, GGT, total bilirubin, APTT, INR, and maximum serum bile acids predisposed pregnant women in the COVID-19 group to severe disease. In addition, baseline concentrations > URL of bile acids, AST, GGT, total bilirubin and mixed type of liver damage on admission correlated with disease exacerbation. Several studies attempted to determine which laboratory parameters were in accordance with the severity of the disease among pregnant COVID-19 patients. Findings of those studies largely mirrored those in the adult nonpregnant population, especially including inflammation parameters and lymphopenia [28].

In the general population there is a substantial data suggesting that severe COVID-19 is associated with higher levels of LFTs than a mild disease, so tracking these markers may allow early identify patients at risk of disease progression [29]. In the meta-analysis intended to compare the laboratory test results between cases of COVID-19 categorized as severe versus non-severe, serum levels of ALT, AST, and TBIL were identified as predictive parameters for ICU admission [30]. In the work conducted by Chen LY patients with both abnormal ALT or AST levels and abnormal ALP or GGT levels presented higher mortality than those with normal LFTs and therefore authors indicated liver damage as an independent prognostic factor of COVID-19 infection [31]. The pattern of elevation is often AST higher than ALT, and this pattern has been associated with disease severity [32, 33]. In fact, present literature suggests that AST and ALT are more commonly elevated than bilirubin or alkaline phosphatase [17, 34]. In the meta-analysis made by Sharma et al. authors revealed that increased AST and ALT values were associated with 3 and 2 times more risk of poor outcomes in COVID-19 patients, respectively [35].

In the pregnancy correlation between abnormal LFTs and disease deterioration is scarce and inconsistent. Fisher SA et al. revealed that transaminitis, elevated PCT and elevated LDH were independently associated with severe to critical disease compared to mild to moderate [36]. In another work written by Choudhary et al. SARS-CoV-2 the infected pregnant women with liver dysfunction manifested in higher serum levels of ALT, AST and TBIL have been documented to experience heightened inflammation, more severe disease, increased morbidity, and higher mortality rates compared to those without liver impairment [23]. Can et al. indicated that liver injury in hospitalized COVID-19 pregnant patients was associated with 3.5-fold risk of disease aggravation and prolonged hospital stay. Moreover, the authors concluded that drug use was the most crucial risk factor for liver injury during hospitalization [21]. No statistical differences in disease aggravation were outlined in work comparing pregnant COVID-19 patients with and without liver damage described as elevation of AST, ALT or TBIL. This work however, is characterized by small sample [37].

Our results indicate that the elevated levels of BA of SARS-CoV-2 positive patients is involved in progression to severe clinical stage of the disease. In the non-pregnant population, serum BA concentration is influenced by various factors, such as BA secretion, intestinal peristalsis, gut flora, blood circulation and absorption in the liver [38] SARS-CoV-2 infection in cholangiocytes initiates an inflammatory response leading to functional impairment and cell breakdown, affecting bile acid release, transport and BA serum levels. This could lead to an amplified inflammatory response in the liver-gut axis, which may be linked to the clinical advancement of COVID-19. Likewise our study, one made by Piñol-Jiménez FN et al. suggests that serum bile acid might be toxic at levels ≥ 10.1 µmol/L, and at such levels is involved in the inflammatory process and predisposed SARS-CoV-2 positive patients to severe course of illness [39]. We believe this indicates the importance of monitoring bile acid serum concentration and treatment protocol including ursodeoxycholic acid in hospitalized pregnant COVID-19 patients.

### LFTs and obstetrics outcomes

The univariate logistic regression showed that AST > URL, ALT > URL and hepatotoxic type of liver injury predisposed pregnant COVID-19 women to poorer obstetric outcomes as measured by incidence of preterm labor and birth weight < 2500 g. In addition, AST > URL and hepatotoxic liver injury type increased the likelihood of low APGAR score at 1 min and the incidence of green amniotic fluid.

The numerous reports in the literature suggest the increased morbidity and mortality in pregnant patients with COVID-19 [13, 40]. Increased rates of perinatal complications posed another threat to SARS-CoV-2 infected pregnant women. In a recent study by Choudhary et al., they compared obstetric outcomes of COVID-19 pregnant women with (*n* = 107) and without (*n* = 142) liver damage. The research revealed no association between liver injury in COVID-19 and obstetric outcomes. However, they did note a higher risk of complications, including postpartum hemorrhage, the requirement for blood transfusion, and sepsis [23]. Furthermore, the study indicated a trend towards preterm birth and cesarean section in the group with liver damage, potentially arising from the exacerbation of underlying conditions like preeclampsia, ICP, or diabetes, which were not excluded [23].

## Conclusion

The hepatic damage in pregnant women with COVID-19 is a common, mild, transaminase-dominant, or mixed type of injury, and often correlates with elevated inflammatory markers. SARS-CoV-2 test should be performed as a part of differential diagnosis in elevated LFTs. Although BA serum levels were commonly elevated, they seems to be clinically irrelevant in terms of pregnancy outcomes. At the initial presentation SARS-CoV-2 infected pregnant women with mixed type of liver injury were more prone to disease progression toward greater clinical severity, probably due to already existing inflammatory effect on various liver cells. Cytotoxic type of hepatic disfunction, especially elevated AST however predisposed to poor obstetric outcomes.

## Data Availability

The datasets generated and/or analyzed during the current study are not publicly available due to our policy but are available from the corresponding author upon reasonable request.
